# Vitamin C further improves the protective effect of GLP-1 on the ischemia-reperfusion-like effect induced by hyperglycemia post-hypoglycemia in type 1 diabetes

**DOI:** 10.1186/1475-2840-12-97

**Published:** 2013-06-27

**Authors:** Antonio Ceriello, Anna Novials, Emilio Ortega, Silvia Canivell, Gemma Pujadas, Lucia La Sala, Loredana Bucciarelli, Maurizio Rondinelli, Stefano Genovese

**Affiliations:** 1 Diabetes and Endocrinology, Insititut d’Investigacions Biomèdiques August Pi i Sunyer (IDIBAPS), Centro de Investigación Biomédica en Red de Diabetes y Enfermedades Metabólicas Asociadas (CIBERDEM), Hospital Clínic Barcelona, C/Rosselló, 149-153, Barcelona 08036, Spainn; 2 Department of Cardiovascular and Metabolic Diseases, IRCCS Gruppo Multimedica, Sesto San Giovanni, MI, Italy

**Keywords:** Diabetes Mellitus, Hypoglycemia, Hyperglycemia, Endothelial Dysfunction, Oxidative Stress, GLP-1

## Abstract

**Background:**

It has been reported that hyperglycemia following hypoglycemia produces an ischemia-reperfusion-like effect in type 1 diabetes. In this study the possibility that GLP-1 has a protective effect on this phenomenon has been tested.

**Methods:**

15 type 1 diabetic patients underwent to five experiments: a period of two hours of hypoglycemia followed by two hours of normo-glycemia or hyperglycemia with the concomitant infusion of GLP-1 or vitamin C or both. At baseline, after 2 and 4 hours, glycemia, plasma nitrotyrosine, plasma 8-iso prostaglandin F2alpha, sCAM-1a, IL-6 and flow mediated vasodilation were measured.

**Results:**

After 2 h of hypoglycemia, flow mediated vasodilation significantly decreased, while sICAM-1, 8-iso-PGF2a, nitrotyrosine and IL-6 significantly increased. While recovering with normoglycemia was accompanied by a significant improvement of endothelial dysfunction, oxidative stress and inflammation, a period of hyperglycemia after hypoglycemia worsens all these parameters. These effects were counterbalanced by GLP-1 and better by vitamin C, while the simultaneous infusion of both almost completely abolished the effect of hyperglycemia post hypoglycemia.

**Conclusions:**

This study shows that GLP-1 infusion, during induced hyperglycemia post hypoglycemia, reduces the generation of oxidative stress and inflammation, improving the endothelial dysfunction, in type 1 diabetes. Furthermore, the data support that vitamin C and GLP-1 may have an additive protective effect in such condition.

## Background

It has recently been suggested that hypoglycemia may play an important role in vascular complications of diabetes [1].

Hypoglycemia causes oxidative stress [2], inflammation [3] and endothelial dysfunction [4]. Oxidative stress is considered the key player in the pathogenesis of diabetic complications [5]. During hyperglycemia oxidative stress is produced at the mitochondrial level [5], similarly as in hypoglycemia [2]. Therefore, oxidative stress might be considered the common factor linking hyperglycemia, hypoglycemia and vascular complications of diabetes. Consistent with this hypothesis is the evidence that both hyperglycemia [6] and hypoglycemia produce endothelial dysfunction and inflammation through the generation of oxidative stress [4,7]. Both endothelial dysfunction and inflammation are well-recognized pathogenic factors for vascular disease, particularly in diabetes [8].

However, there is evidence that free radical production rises not only during hypoglycemia, but particularly during glucose reperfusion after hypoglycemia [9]. In both mice and cultured neurons, hypoglycemia followed by different concentrations of glucose reperfusion has been linked to a degree of superoxide production and neuronal death that increased proportionally with glucose concentrations during the reperfusion period [9]. Consistently, it has recently been demonstrated that in type 1 diabetes hyperglycemia following hypoglycemia worsens endothelial dysfunction and inflammation through the generation of an oxidative stress [10]. These findings suggest that hyperglycemia following hypoglycemia produces a “reperfusion”-like effect [11].

GLP-1 and its analogues are now being used as therapy in patients with type 2 diabetes [12] in whom a defect of GLP-1 secretion/action in response to the meal has often been reported [13]. However, recently, a possible beneficial effect of GLP-1 analogues in the management of also type 1 diabetes has been suggested [14]. GLP-1 analogues, in addition to their insulin-tropic action, have beneficial effects in protecting pancreatic β-cell function, in suppressing glucagon secretion and in delaying gastric emptying, all characteristics which could be beneficial for the management of type 1 diabetes [14].

Apart from the well-documented incretin effect of glucagon like peptide 1 (GLP-1), its role in the cardiovascular system also arouses interest. GLP-1 effects on the cardiovascular system may include a direct action on the endothelium, where the presence of specific receptors for GLP-1 has been demonstrated [15]. Consistently, GLP-1 has demonstrated to improve endothelial function in diabetes [16,17]. This protective effect should be exerted improving the antioxidant defenses of the endothelium [18] and decreasing oxidative stress generation [17]. Furthermore, GLP-1 seems to have a protective effect during ischemia/reperfusion of the heart [19].

Overall, the situation seems to be more complicated. It has been demonstrated that in type 2 diabetes hyperglycemia induces a resistance to the action of GLP-1 at the level of the β-cell, endothelium and muscle, being the oxidative stress the mediator of such phenomenon [13,17,20].

The aim of this study is to explore if GLP-1 can protect endothelial function and can reduce inflammation when hypoglycemia is recovered with hyperglycemia, and if this protective action is related to a decrease in oxidative stress. Furthermore, to explore a possible appearance of a resistance to GLP-1 action during hyperglycemia post hypoglycemia.

## Methods

### Subjects

Fifteen persons with type 1 diabetes were studied (Table 1). They were treated with multiple daily insulin injections, had normal bedside tests of autonomic function [17] and did not exhibit hypoglycemia unawareness as based on the methods of Gold, et al. [18], as well as had no major macro or micro complications of diabetes. Subjects were excluded from the study if they had a history of at least one major episode of hypoglycemia in the preceding 2 years. All subjects were nonsmokers and had normal blood count, plasma lipids, plasma electrolytes, liver and renal function, and blood pressure. No subject was taking medications known to affect neuroendocrine responses to hypoglycemia or inflammation. Studies were approved by the Ethics Committees of the respective research institutions involved, and all participants gave written informed consent.

**Table 1:** Baseline characteristics of the type 1 diabetic patients

**Sex**	**8M 7F**
Age years	23.2 ± 3.1
BMI Kg/m2	23.9 ± 2.7
Duration of the disease/years	7.6 ± 1.5
HbA1c %	7.8 ± 0.2
HbA1c mmol/mol	61.7 ± 2.1
Resting diastolic blood pressure mm Hg	77.3 ± 1.4
Resting systolic blood pressure mm Hg	116.1 ± 1.6
Total cholesterol mmol/l	4.2 ± 0.3
Triglycerides mmol/l	1.2 ± 0.2
HDL-C mmol/l	1.4 ± 0.2
LDL-C mmol/l	2.2 ± 0.2
FMD %	6.8 ± 0.8
8-iso-PGF2a (pg/ml)	65.5 ± 45.2
Nitrotyrosine μmol/l	0.68 ± 0.03
sICAM-1a (ng/ml)	170.4 ± 10.7
IL-6 (pg/ml)	234.10 ± 14.1

Data are expressed as mean ± SE.

All study patients were asked to avoid any exercise and consume their usual weight-maintaining diet for 3 days before each experiment. All people with type 1 diabetes were asked to perform intensive home blood glucose monitoring and to avoid hypoglycemia for at least 5 days before the study. On the day prior to the study, intermediate or long-acting insulin was discontinued and replaced with injections of regular insulin before breakfast and lunch. Subjects were admitted to the research center the evening before the experiment, at which time the individuals with type 1 diabetes had two intravenous cannulas inserted with 1% lidocaine local anesthesia. One cannula was placed to be used for drawing blood, while the other was placed in the contralateral arm for infusions. All subjects received an evening meal and received a continuous low-dose infusion of insulin to normalize plasma glucose. The insulin infusion was adjusted overnight to maintain blood glucose between 4.4 and 7.2 mmol/l.

### Hypoglycemia experiments

Five different experiments, each following a two-hour period of induced hypoglycemia and lasting for two hours, were planned for each subject in a randomized order: protocol 1, reaching and maintaining normoglycemia; protocol 2, reaching and maintaining hyperglycemia; protocol 3, reaching and maintaining hyperglycemia with simultaneous infusion of vitamin C; protocol 4, reaching and maintaining hyperglycemia with simultaneous infusion of GLP-1; protocol 5, reaching and maintaining hyperglycemia with simultaneous infusion of vitamin C plus GLP-1. The randomization was carried out using a computer-generated, random-number sequence. Each subject underwent the experiments at two-weeks or longer intervals.

In each experiment, the subjects fasted overnight for 10 hours. In all experiments, after an initial steady state period of 120 min, a 2 h hyperinsulinemic hypoglycemic clamp was performed, with a primed constant (9.0 pmol • kg − 1 • min − 1) infusion of insulin (Actrapid, NovoNordisk, Copenhagen, Denmark). The rate of glucose decrease was controlled (~0.08 mmol/min), and glucose nadir (2.9 mmol/l) was achieved using a modification of the glucose clamp technique. During the clamp period, plasma glucose was measured every 5 min, and a 20% dextrose infusion was adjusted so that plasma glucose levels were held constant at 2.9 ± 0.1 mmol/l [19]. Potassium chloride (20 mmol/l) was infused during the clamp to reduce insulin-induced hypokalemia. After this period of induced hypoglycemia, common to all the experiments, 20% dextrose infusion was adjusted in order to reach and maintain normoglycemia (4.5 mmol/l), or to reach and maintain hyperglycemia (15 mmol/l), or to reach and maintain hyperglycemia with the simultaneous infusion of vitamin C (Bracco, Milan, Italy) [30 mg/min, [10]], or GLP-1 (Synthetic GLP-1 (7–36) amide, PolyPeptide Laboratories, Wolfenbuttel, Germany) [0.4 pmol Kgˉ^1^ min ˉ^1^[21]], or both, for additional 120 min.

At baseline and after 2 and 4 hours, blood samples were withdrawn for biochemical assays (glycemia, plasma nitrotyrosine and plasma 8-iso prostaglandin F2alpha (8-iso-PGF2a), both markers of oxidative stress, ICAM-1a and IL-6, markers of inflammation), while endothelial function was measured by flow mediated dilation (FMD).

### Biochemical and clinical measurements

Cholesterol, triglycerides, HDL-C, LDL-C and plasma nitrotyrosine were measured according to [22]. Plasma glucose was measured by the glucose-oxidase method, HbA1c by HPLC. Plasma 8-iso-PGF2a (Cayman Chemical, Ann Arbor, Michigan, U.S.A.), sICAM-1 (British Bio-technology, Abington, Oxon, U.K.) and IL-6 (R&D Systems, Minneapolis, MN, USA), were determined with commercially available kits.

### Endothelial function

Endothelial function was evaluated measuring the FMD of the brachial artery [10,17]. At the end of each test, the subjects laid quietly for 15 min. Then, sublingual nitroglycerin (0.3 mg) was administered and 3 min later the last measurements were performed. Response to nitroglycerin was used as a measure of endothelium-independent vasodilation.

### Statistical analysis

The sample size was selected according to previous studies [3-10].

Data are expressed as means ± SE. The Kolmogorov-Smirnov algorithm was used to determine whether each variable had a normal distribution. Comparisons of baseline data among the groups were performed using unpaired Student’s *t*-test or Mann–Whitney *U*-test, where indicated. The changes in variables during the tests were assessed by one-way ANOVA with repeated measures or Kolgorov- Smirnof test, where indicated. If differences reached statistical significance, post hoc analyses with two-tailed paired *t* test or Wilcoxon signed rank test for paired comparisons were used to assess differences at individual time periods in the study. Statistical significance was defined as p < 0.05.

## Results

**Figure 1:**
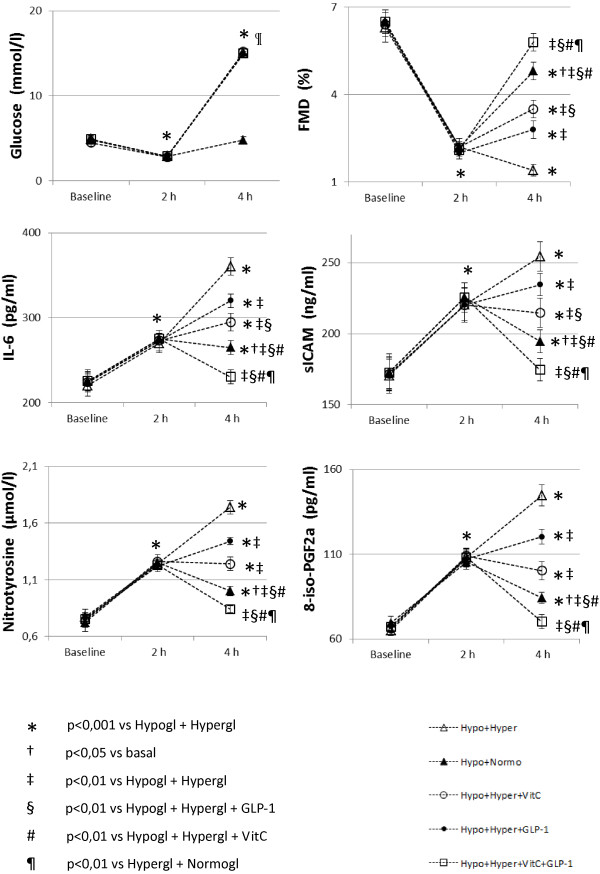
Glycemia, sICAM-1, flow-mediated dilation (FMD), nitrotyrosine, IL-6 and 8-iso-PGF2a in type 1 diabetes during the experiments.

Similarly to previous studies [4-6], after 2 h of hypoglycemia FMD significantly decreased, while sICAM-1, 8-iso-PGF2a, nitrotyrosine and IL-6 significantly increased, compared to basal values (Figure 1).

As already reported [10], the way to recovery from hypoglycemia, had a dramatic different effect when obtained reaching normoglycemia or hyperglycemia.

Similarly to our previous study, after 2 h of recovering in normoglycemia, FMD was still significantly decreased, while sICAM-1, 8-iso-PGF2a, nitrotyrosine and IL-6 were still significantly increased, compared to basal values (Figure 1). However, after 2 h of recovering in hyperglycemia, FMD decreased, and sICAM-1, 8-iso-PGF2a, nitrotyrosine and IL-6 were increased even more significantly compared to basal as well as to the recovery in normoglycemia (Figure 1).

When the recovery in hyperglycemia was accompanied by the simultaneous infusion of GLP-1, all these phenomena were significantly attenuated: FMD decreased less, while sICAM-1, 8-iso-PGF2a, nitrotyrosine and IL-6 were less increased (Figure 1). Vitamin C was even more effective than GLP-1 in decreasing all these phenomena (Figure 1). Finally, the simultaneous infusion of GLP-1 and vitamin C completely abolished the deleterious effects of hyperglycemia following the hypoglycemia (Figure 1).

Endothelial independent vasodilatation was not affected in any of the experiments. The amount of insulin infused during all the experiments was similar (AUC 75,200 ± 350 vs 75,320 ± 370 vs 75,140 ± 350 vs 75,280 ± 280 vs 75,370 ± 270 pmol).

## Discussion

This study confirms our previous results that recovering hypoglycemia with hyperglycemia worsens endothelial function and inflammation, being oxidative stress generation the possible cause of this effect [10]. We and others have previously suggested that hyperglycemia following hypoglycemia generates an ischemia/reperfusion like situation, generating an oxidative stress [9,10]. The role of the oxidative stress is also supported by the data obtained infusing vitamin C.

In this study, for the first time, we report that GLP-1 can partly counterbalance this phenomenon. Recently, the crucial role of GLP-1 in cardiovascular disease has been suggested by both preclinical and clinical studies [19]. It has been reported that GLP-1 may have cardio-protective effects, being ale of reducing the ischemia/reperfusion injury and also cardiac dysfunction in various animal models and humans [19,23]. Several mechanisms may explain the effect of GLP-1 in reducing the damage induced by the ischemia/reperfusion and, between them, the possibility that GLP-1 can reduce oxidative stress and can increase antioxidants, leading to decreased apoptosis [24,25]. Therefore, it is reasonable that, in our study, GLP-1 should, reducing oxidative stress generation, improve endothelial dysfunction and inflammation generated by hyperglycemia following hypoglycemia.

It is worthy of interest that vitamin C was more efficacious than GLP-1 in counterbalancing the effects of hyperglycemia post hypoglycemia and that only when both vitamin C and GLP-1 were simultaneously infused this effect is quite completely abolished. It is important to underline how vitamin C and GLP-1 act as antioxidants. Vitamin C has a scavenger action, which means that it “captures” the free radicals when they are already generated [24-26]. GLP-1, as other compounds, increases the intracellular antioxidant defenses. This means that they can cooperate on the global oxidative stress generated, because they have a complimentary action. The data show that, even vitamin C has been infused at the supposed maximal antioxidant effect, the alterations in endothelial function and inflammation are still persistent even improved. This means, in our opinion, that some “oxidative stress” still escapes from the action of vitamin C. In this view, it is reasonable that adding another antioxidant, such as GLP-1, which works as antioxidant in a very different way, the effect on oxidative stress and on its related alteration can be further improved.

A possible influence of insulin by itself on the results cannot be excluded, particularly because it has recently been reported that GLP-1 enhances the vasodilator effect of insulin [27]. However, in our experiments, the amount of insulin infused was the same during all the experiments, therefore, the role of insulin in the study has certainly been minimized. In the same paper by Tesauro et al. [27], GLP-1 was unable to enhance the effect of vitamin C on endothelial dysfunction. However, our experimental conditions are very different. We studied fifteen people with type 1 diabetes, while the study of Tesauro was focused on only five people with the metabolic syndrome [27]. Moreover, as underlined by the same authors, no control study, with vitamin C alone, were performed.

It is also worthy of interest the recent report demonstrating that three months of exenatide therapy had similar effects on microvascular endothelial function, markers of inflammation, oxidative stress, and vascular activation, as metformin, in patients with obesity and pre-diabetes [28], which could question the specific role of GLP-1 analogues in preserving endothelial function. However, it is important to underline that also metformin, as GLP-1 [29], has an antioxidant power. Therefore this similarity of the effect between the two compounds could may explain the results of this study.

In our opinion this report has also important practical implications. The risk of a cardiovascular disease in type 1 diabetes is very high [30], and the role of the oxidative stress seems to very relevant in the pathogenesis of these complications in type 1 diabetes [5]. The interest in understanding if hypoglycemia, which produces an oxidative stress [1,2], is a risk factor for cardiovascular disease in type 1 diabetes is increasing, even the epidemiological data are quite conflicting [31-33]. We have already suggested the hypothesis that the way in which recovery from hypoglycemia takes place, because may increase the oxidative stress, may condition the cardiovascular outcomes [10]. It is currently suggested that GLP-1 analogues could be helpful in the management of type 1 diabetes, not only because can contribute to improve the metabolic control and to reduce insulin requirement [14], but also because GLP-1 can protect type 1 diabetes from hyperglycemia and hypoglycemia-induced oxidative stress, inflammation and endothelial dysfunction [34]. Our data suggest that another potential good reason to use GLP-1 analogues in the management of type 1 diabetes might be related to their potential to reduce oxidative stress, generated during hyperglycemia following hypoglycemia.

In conclusion, this study confirming the possibility that vitamin C counterbalances the deleterious effects of hyperglycemia post-hypoglycemia underlines the role of the oxidative stress in this phenomenon. At the same time, this study showing that GLP-1 can counterbalance the deleterious effect of the ischemia/reperfusion induced by the recovery from hypoglycemia with hyperglycemia, on oxidative stress generation, inflammation and endothelial dysfunction, supports the usefulness of GLP-1 and its analogues in the management of type 1diabetes.

## Abbreviations

GLP-1: Glucagon like peptide 1; 8-iso-PGF2a: 8-iso prostaglandin F2alpha; ICAM-1: Intercellular adhesion molecule-1; IL-6: Interleukin 6; FMD: Flow mediated dilation.

## Competing interests

The authors do not have any conflict of interest to disclose.

## Authors’ contributions

AC, contributed to: researched data, discussion, wrote manuscript, reviewed/edited manuscript. AN contributed to: researched data, discussion, reviewed/edited manuscript. EO, contributed to: researched data, discussion, reviewed/edited manuscript. SC, contributed to: discussion, reviewed/edited manuscript. GP contributed to: researched data, discussion. LLS contributed to: researched data, discussion. LB, contributed to: researched data, discussion, reviewed/edited manuscript. MR, contributed to: discussion, reviewed/edited manuscript. SG, contributed to: discussion, reviewed/edited manuscript. All authors read and approved the final manuscript.
